# Post kidney transplant hematologic abnormalities and association of post-transplant anemia with graft function

**DOI:** 10.12688/f1000research.144124.2

**Published:** 2024-09-26

**Authors:** Sindhura Lakshmi Koulmane Laxminarayana, Shreya Jayaram, Shilna Muttickal Swaminathan, Ravindra Prabhu Attur, Dharshan Rangaswamy, Indu Ramachandra Rao, Mohan V Bhojaraja, Srinivas Vinayak Shenoy, Shankar Prasad Nagaraju

**Affiliations:** 1 Department of Pathology, Kasturba Medical College, Manipal, Manipal Academy of Higher Education,, Manipal, Karnataka, 576104, India; 2 Department of Nephrology, Kasturba Medical College, Manipal, Manipal Academy of Higher Education, Manipal, Karnataka, 576104, India

**Keywords:** chronic kidney disease, renal transplantation, post-transplant anemia, renal allograft rejection, post-transplant infections

## Abstract

**Background:**

Haematological abnormalities following renal transplantation are frequently observed and have a significant effect on survival and graft outcomes. The pattern of haematological abnormalities varies globally. Few studies have been conducted in Asian countries. We aimed to evaluate the patterns of haematological abnormalities in post-transplant recipients in our center during the first year after post-renal transplant and the association of post-transplant anemia with graft function.

**Methods:**

This single-center retrospective study was conducted on renal transplantation recipients between 2014 and 2019. The study included all patients who received kidney transplants from live/cadaveric donors and had follow-up data collected up to 12 months after the transplant. The outcome studied was the prevalence of haematological abnormalities and the association between post-transplant anemia (PTA) and graft function in post-transplant recipients.

**Results:**

A total of 106 renal transplant recipients were included in the study. The prevalence of PTA was 98% in the first week, 75% at one month, 35% at three months, 32% at six months, and 27% at 12 months. The other cytopenia cases were leukopenia (43.4%), thrombocytopenia (33.2%), and pancytopenia (15.1%). Post-transplant erythrocytosis was observed in 17.9% of patients. 18 patients with severe PTA in the first week of transplant had significant allograft dysfunction (p=0.04). Patients with and without PTA had similar graft functions at six and 12 months (p=0.50).

**Conclusions:**

Haematological abnormalities are common in renal transplant recipients. PTA is highly prevalent during the first week and improves over time. Other haematological abnormalities observed were leukopenia, thrombocytopenia, pancytopenia, and post-transplant erythrocytosis. Leucopenia was primarily drug-induced, and thrombocytopenia and pancytopenia were frequently caused by infections in our cohort. Additionally, severe PTA was significantly associated with graft dysfunction in the first week post-transplant, whereas similar graft function was observed at 6 and 12 months post-transplant, irrespective of the presence or absence of PTA.

## Introduction

Chronic kidney disease (CKD) is recognized as a significant cause of morbidity and mortality and globally around 843.6 million individuals are affected by this condition ^1^ End-stage kidney disease (ESKD) is a growing global health concern and has a substantial impact on the quality of life ^2^; renal transplantation represents the ultimate treatment modality for patients with stage 5 CKD or ESKD ^3^; however, renal transplantation improves the quality of life and is susceptible to various post-transplant complications such as metabolic disturbances, life-threatening infections, malignancy, and haematological disturbances. Haematological abnormalities are frequently observed in renal transplant recipients and can lead to severe complications in transplant recipients if not recognized early. Common post-transplant blood disorders include post-transplant anemia (PTA), post-transplant cytopenia (PTC), including leukopenia, thrombocytopenia, pancytopenia, post-transplant lymphoproliferative disorder (PTLD), and post-transplant erythrocytosis (PTE). ^4^^,^^5^ A better understanding of the prevalence and etiology of haematological abnormalities may help effectively address and mitigate these complications in our day-to-day practice.

Anemia is a frequently observed complication in CKD patients, and its prevalence is higher in renal transplant patients and varies with time after transplantation. ^6^ Earlier studies have shown that in post-renal transplantation, there is a biphasic pattern of anemia, and the incidence rate is higher, about 76% at the time of transplantation, 21% in 1 ^st^ year, and 36% in the 4 ^th^ year of transplantation. ^7^ Several factors are linked to PTA, such as infection, iron deficiency, declining renal function, recurrent transplantation, and administration of immunosuppressive medications. Furthermore, long-term follow-up studies (nearly 4 years) have demonstrated that baseline anemia serves as an effective predictor of mortality and graft failure in kidney transplant recipients, and anemia can be considered as an important risk factor for graft rejection/dysfunction in these patients. ^6^^,^^8^

Other common haematological abnormalities, such as leukopenia, thrombocytopenia, and pancytopenia, change with the type of induction agent, maintenance immunosuppressive drug, and dose used, which varies across centers. Over the past few decades, the implementation of CMV prophylaxis has led to a reduction in both its prevalence and associated haematological abnormalities. The estimated incidence of leukopenia in kidney transplant patients ranges from 10–55%. ^9^ There is also a high rate of thrombocytopenia following renal transplantation in the first year, with the majority of recipients experiencing the lowest platelet counts within the first three months after the transplant ^10^ which causes an oxygen deficit and immune system dysfunction that have a broad impact on the body. ^11^ Post-transplant erythrocytosis is usually observed in 10–15% of patients ^12^ and is typically evident within the first year of transplant. These haematological abnormalities can indicate various underlying causes, leading to a therapeutic dilemma in determining the most appropriate course of treatment. Against this backdrop, the present study aimed to evaluate haematological abnormalities in post-renal transplant patients. Additionally, we studied the association between the first week post-transplant severe anemia and graft dysfunction, and compared graft function at both early (6 months) and late (12 months) post-transplant anemia.

## Methods

### Study design

This retrospective single-center observational study included 106 renal transplant recipients who underwent kidney transplantation at Kasturba Medical College, Manipal, Manipal Academy of Higher Education, Manipal, India.

### Inclusion criteria

**Figure 1.**
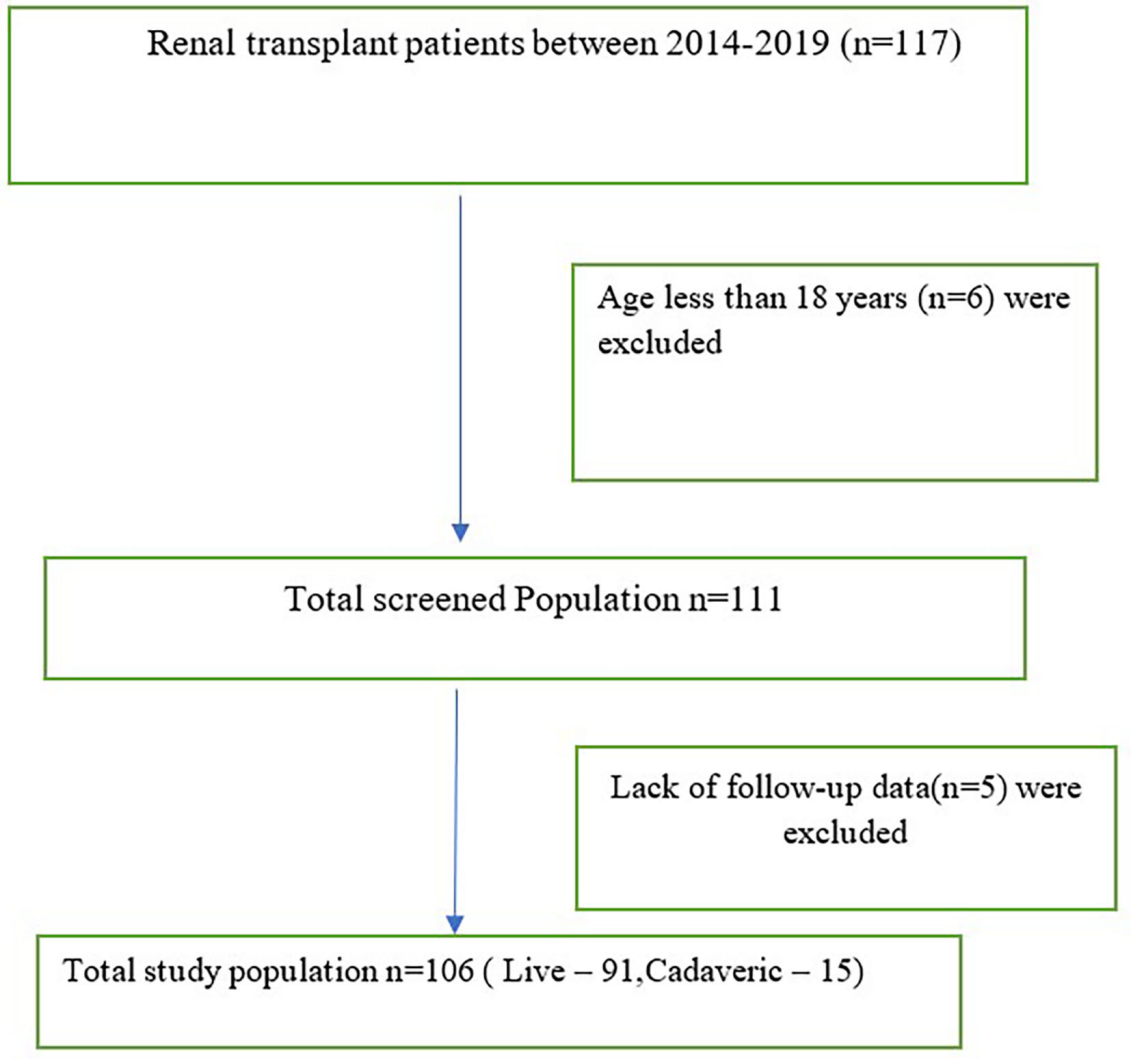
Flow diagram of the study population.

The study included all patients who underwent kidney transplants from live/cadaveric donors between January 2014 and December 2019 ( Figure 1).

### Exclusion criteria

Renal recipients below the age of 18 years and those without available follow-up data for a minimum of one year were excluded ( Figure 1).

### Study procedure

This was a retrospective case-record-based study. Medical records were reviewed and clinical and laboratory parameters were tabulated. The research conducted in this study adhered to the principles outlined in the Declaration of Helsinki and was approved by the Kasturba Medical College and Kasturba Hospital Institutional Ethics Committee (IEC 542/2019) on 6 ^th^ September 2019. A waiver of consent was granted as per our institutional ethics committee due to the retrospective nature of the study. Baseline demographic data and laboratory data, including complete blood counts (CBC) and serum creatinine levels, were collected from the pre-transplant period (<1 week). The follow-up details of CBC and serum creatinine were collected at the post-transplant first week, one month, three months, six months, and 12 months and tabulated, and the prevalence of PTA was studied. The time taken for normalization of the hemoglobin level was also documented.

Other haematological abnormalities including leukopenia, thrombocytopenia, and pancytopenia were also collected, and the type of immunosuppression was noted. The donor details were also collected. The association between the first week post-transplant severe anemia and graft dysfunction was analyzed, and graft function was compared at both early (six months) and late (12 months) post-transplant anemia.

### Definitions

Anemia: “Hemoglobin level less than 12 g/dL in women and less than 13 g/dL in men according to the World Health Organization (WHO) criteria and KDIGO”. ^13^

Leukopenia: WBC count less than 4000/μL. ^14^

Thrombocytopenia: platelet count less than 150,000/μL. ^14^

The presence or absence of leukopenia and thrombocytopenia were defined at post-transplant time points of <6 months and >6 months.

Acute and chronic rejections were confirmed by biopsy. Delayed graft function was characterized by the need for at least one dialysis treatment during the first week following transplantation. Slow graft function was characterized by a 30% creatinine reduction ratio on day two and 70% creatinine reduction ratio on day 7. ^15^

### Outcome measures

The primary outcome studied was the prevalence of the most common haematological abnormalities in post-renal transplant patients.

The secondary outcomes studied were the association between the first week post-transplant severe anemia and graft dysfunction and the comparison of graft function between patients with and without PTA at six months and 12 months post-transplant.

### Statistical methods

Discrete variables are represented as frequencies and percentages. Continuous data are shown as mean±SD. An unpaired T-test or Wilcoxon signed-rank test was performed to compare parameters between different time points. Normality was assessed using the Kolmogorov-Smirnov test. A chi-squared test was performed to assess the association between the parameters. Statistical significance was set at P<0.05. The data were analyzed using SPSS version 29.

## Results

The demographics and clinical characteristics of the renal transplant recipients are shown in Table 1 and in the underlying data. ^16^^,^^17^ The dose of basiliximab used was 20 mg intravenously on 0 and 4 ^th^-day post-operation. The ATG dose used varied from to 1-3 mg/kg at the nephrologist’s discretion. All patients received 500 mg methylprednisolone along with induction therapy. All patients were treated with triple immunosuppression (Tacrolimus, Mycophenolate, and steroids) as per KDIGO guidelines. The Tacrolimus dose used was 0.08 mg/kg/day in two divided doses to aim for 10-12 mcg/mL trough level in first month, 6-10 mcg/mL up to 3 months and then onwards 3-6 mcg/mL. The Mycophenolate mofetil dose used varied from 1.5 gram to 2 gram per day based on the nephrologist’s decision and body weight of the patient.

**Table 1.** Demographics and clinical characteristics of the study participants.

Variables	Renal transplant recipients (n=106)
Donor age, *In years*	46.6±10.3 ^a^
Recipient age, *In years*	36.9±10.8 ^a^
Donor gender ( *n, %)*	
Male	24(22.5)
Female	82(77.2)
Recipient gender ( *n, %)*	
Male	91(85.8)
Female	15(14.2)
BMI	21.0±4.2 ^a^
Donor type ( *n, %)*	
Live	91(85.8)
Cadaveric	15(14.2)
Pre transplant Hb(g/dl)	9.4±1.7 ^a^
Pre-transplant WBC count -*10/microL	7.2±3.1 ^a^
Pre-transplant platelet count -*10/microL	205.8±32.4 ^a^
**Induction therapy (n (%))**	
Basiliximab	84(79.2)
Antithymoglobulin	22(20.7)

^a^ Mean±SD; BMI, Body mass index.

The haematological abnormalities of patients post-transplantation are shown in Table 2.

**Table 2.** Post-transplant haematological abnormalities and allograft rejection status.

Clinical variables	Renal transplant recipients (n=106)
Post-transplant anemia	
1 week	104(98%)
1 month	80(75%)
3 months	38(35%)
6 months	34(32%)
12 months	29(27%)
Post transplant leukopenia	46(43.4%)
Post transplant thrombocytopenia	33(31.2%)
Post transplant pancytopenia	16(15.1%)
Post transplant lympho-proliferative disorder	0
Post transplant erythrocytosis	19(17.9%)
Graft dysfunction	
Slow and delayed graft function	11(10.3%)
Acute rejection	19(17.9%)
Chronic rejection	6(5.6%)

### Post-transplant anemia

**Figure 2.**
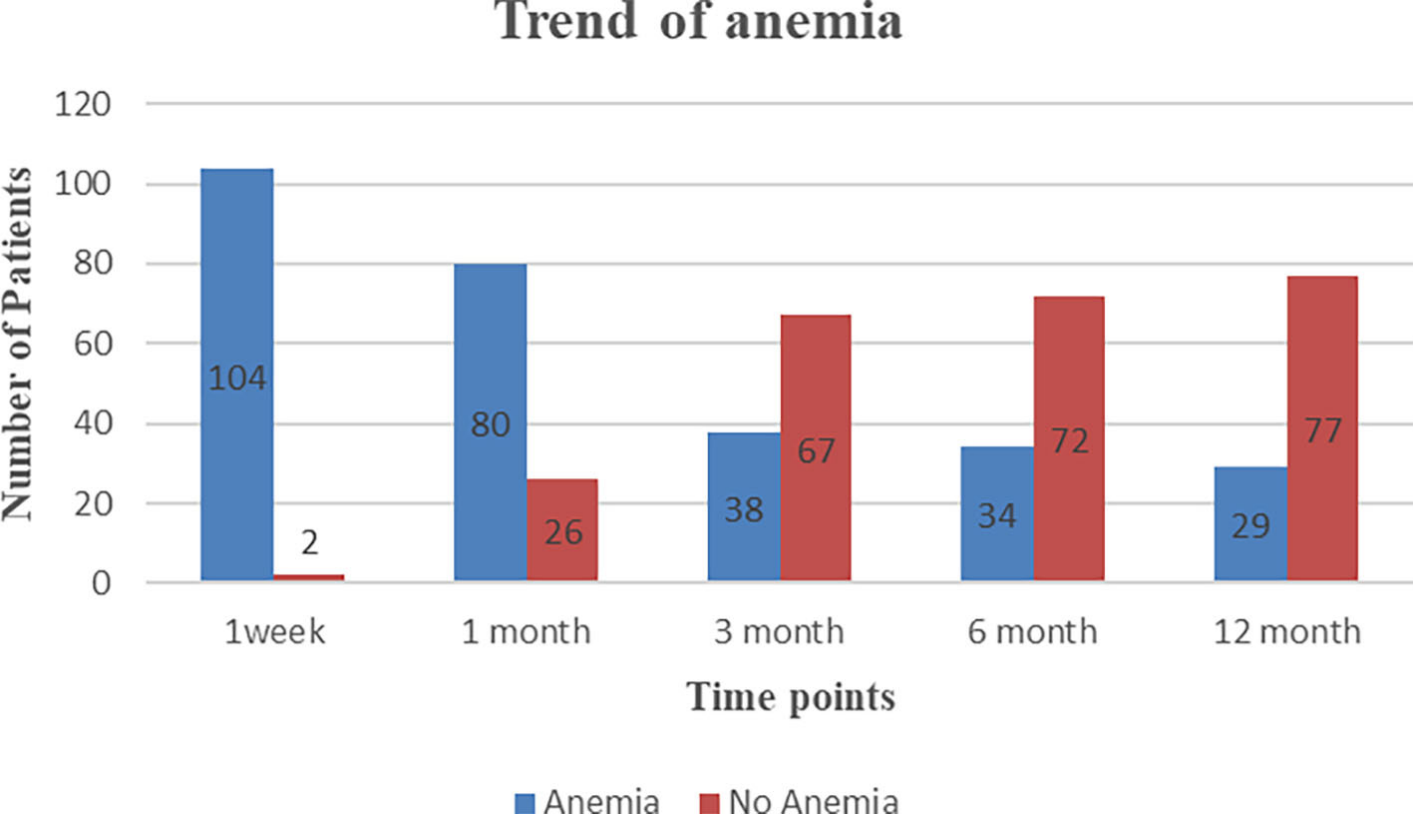
Trend of post-transplant anemia.

PTA is highly prevalent in our study population and has improved over time. Incidence of pre-transplant anemia was also high and reported in 101 patients (95%). As shown in Figure 2, the prevalence of post-transplant anemia showed a trend of 98% during the first week, 75% at one month, 35% at three months, 32% at six months, and 27% at 12 months post-transplantation. Further on stratification of severity of anemia, mild anemia was found to be common among PTA ( Table 3). Additionally, 41.7% of severe anemia cases were reported within the first week after transplantation. None of the patients had features of microangiopathic hemolytic anemia.

**Table 3.** Stratification of post-transplant anemia.

Time of events	Severity of anemia		
	Severe (Hb <10 g/dl)	Mild (Hb 10-12/13 g/dl)	No anemia (Hb >12/13 g/dl)
Post 1 week	45(41.7)	59(54.6)	2(1.9)
Post 1 month	39(36.2)	41(42.7)	26(24.5)
Post 3 month	18(16.7)	20(18.8)	68(64.1)
Post 6 month	12(11.3)	22(20.7)	72(67.9)
Post 12 month	8(7.5)	21(19.1)	77(72.6)

**Figure 3.**
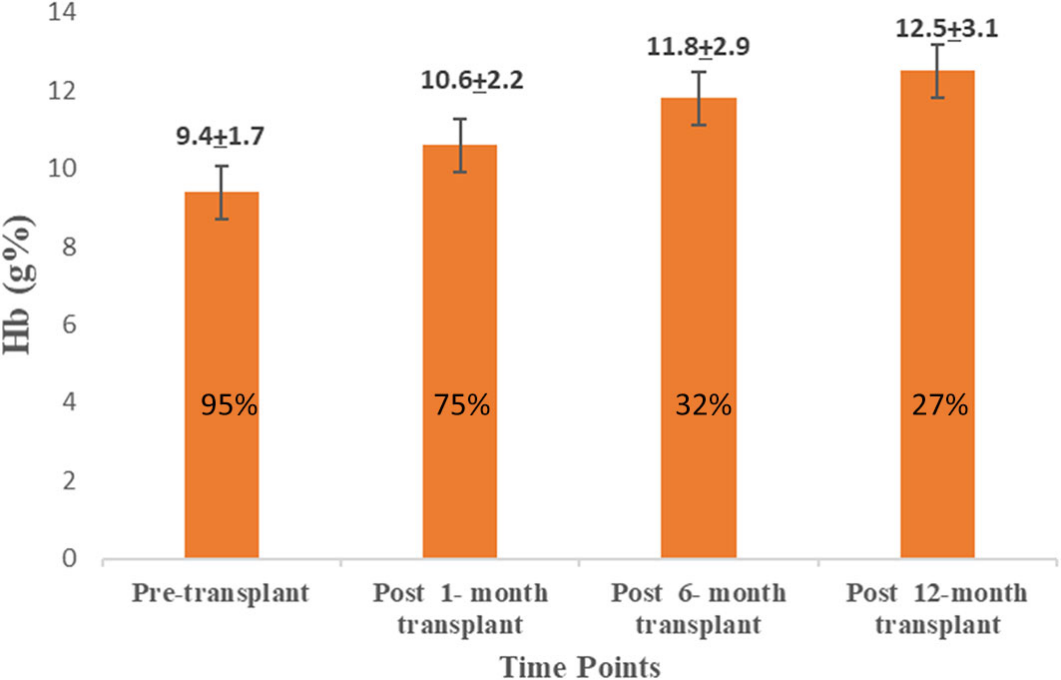
Comparison of pre-transplant and post-transplant hemoglobin levels.

The pre-transplant Hb level was 9.4±1.7, and post-transplant there was a significant improvement in the Hb levels at one month (10.6±2.2), six months (11.8±2.9), and 12 months (12.5±3.1), which was significant when compared to pre-transplant levels (p<0.001) ( Figure 3).

### Leukopenia

Leukopenia was observed in 35(76%) patients in first 6 months and 11(23.9%) after 6 months accounting for overall prevalence of 43%. The mean leucocyte count was 3.2 ± 1.2 ×10 ^3^/μL. Neutropenia was reported in 16 patients (34.7%) and 10 patients of them required G-CSF for recovery. The primary cause for leukopenia was drug induced (93%) and CMV was the second most common cause (7%).

### Isolated thrombocytopenia

Thrombocytopenia occurred in 31.2% of the patients in the overall study group, which was observed in 20(60.2%) patients in first 6 months and 13(39.3%) developed after 6 months. The mean platelet count was 70 ± 12 × 10 ^3^/μL. The majority of cases were attributed to bacterial infections (84.5%), followed by fungal infections (9.2%), and CMV infections (6.3%).

### Pancytopenia

We observed that 16(15.1%) patients had pancytopenia. The major cause was drug-induced 12(75.6%) followed by CMV in 3 patients (18.6%) and bacterial infections were found in one patient (6%). G-CSF was required in 10 patients (62%) for pancytopenia recovery.

During the cytopenia episodes, all patients underwent testing for CMV infection (CMV PCR). All patients were on mycophenolate mofetil (1.5-2.00 g/day). All transplant recipients received sulfamethoxazole and trimethoprim (400 mg + 80 mg) as bacterial infection prophylaxis for first year and Valgancyclovir 450 mg once daily as CMV prophylaxis for first 6 months. During leukopenia/pancytopenia episodes all the three drugs were withheld until count recovery. The mycophenolate mofetil was restarted after the count recovery at lower dose. The Valgancyclovir and trimethoprim sulfamethoxazole were restarted.

### Erythrocytosis

Nineteen patients (17.9%) had erythrocytosis. The incidence was higher in males (72%), and the mean hematocrit count was 56±13%.

### Graft dysfunction and severe anemia at first-week post-transplant

In the first week post-transplant, slow or delayed graft function was observed in 11 recipients (22.3%), 19 patients (39.5%) had acute rejection. Specifically, 15 cases were diagnosed as cell-mediated rejection, while four cases were identified as antibody-mediated rejection on biopsy. Severe post-transplant anemia in the first week was significantly associated with allograft dysfunction (SGF, DGF, and acute rejection) (p=0.04) ( Table 4).

**Table 4.** Association between graft dysfunction (SGF+DGF/acute rejection) and severe anemia. SGF: Slow graft function; DGF, Delayed graft function.

Variables	Severe anemia		P value
Kidney allograft dysfunction	No	Yes	
Yes	12	18	0.04 *
No	14	05	

* Statistically significant.

### Graft function in patients with and without PTA at early (six months) and late (12 months) post-transplant

At six and 12 months, mean serum creatinine levels for patients with PTA were 1.6±0.6 mg/dl with eGFR 68.0±19.3 mL/min/1.73 m ^2^ and 1.7+0.5 mg/dl with mean eGFR 74.2±22 mL/min/1.73 m ^2^. While for patients without PTA, the mean serum creatinine levels were 1.4±0.5 mg/dl with eGFR 64.3±22 mL/min/1.73 m ^2^ and 1.3±0.4 mg/dl with mean eGFR 64.8±21 mL/min/1.73 m ^2^ at six and 12 months respectively. Although the serum creatinine level was slightly higher in patients who had PTA, it was not statistically significant and was comparable between the groups (p=0.50), implying that PTA did not have any effect on graft function in our cohort at six and 12 months after renal transplant.

## Discussion

Following renal transplantation, in addition to common metabolic and infectious complications, patients also encounter haematological abnormalities. ^18^ In our study population, the most prevalent blood-related complication was PTA. Renal transplant patients experience anemia at various periods after transplantation, and the cause of anemia differs with time. post-transplant anemia is usually classified as early anemia, which occurs at or less than six months post-transplantation, with a prevalence rate of 50%, and late anemia, which occurs six months post-transplantation, with a prevalence rate of 23–35%. ^19^ Few studies have defined PTA occurring in the first week of transplant as immediate post-transplant anemia. ^20^

In our study, the prevalence of anemia was 98% in the first week, 75% at one month, 35% at three months, 32% at six months, and 27% at 12 months after transplantation, and there was a noticeable downward trend in prevalence over the study period. Similarly, Poesen *et al*. ^21^ observed a 98% prevalence during the immediate post-transplant period, and Talwar *et al*. ^20^ found that 100% of recipients had PTA in the first week and 76.7% in the third month post-transplantation. Additionally in a retrospective study by Gafter-Gvili *et al*. ^22^ the prevalence of PTA at six months was 51.3% and at two years it was 36%. The notable differences in anemia prevalence may be due to various diagnostic criteria, varied causes, and different clinical practices across different healthcare centers worldwide, ^23^ such as iron deficiency as a result of iron store depletion during pre-transplantation, perioperative blood loss, and malnutrition. ^24^ Late PTA is usually associated with decreased graft function and occurrence of renal insufficiency. ^25^

In the present study, there was a significant improvement in the hemoglobin level at post-transplant one, six and 12 months as compared to the pre-transplant and it was significant (p<0.001), which was consistent with Radoui *et al*. ^26^ who reported the pre-transplant Hb improvement from 10.2 g/dl at one month to 12.8 g/dl at sixth month and then to 13.2 g/dl at one year.

The broad causes of cytopenia may be drug toxicity/interactions, immune reactions, or viral infections. In the present study, the prevalence of post-transplant leukopenia (PTL) is 43.4%, which was consistent with the findings of Smith *et al*. ^27^ were reported 47.5%. In our study population, drug induced was identified as the primary agent associated with leukopenia, which is consistent with Zafrani et al. ^9^ In contrast, Max *et al*. reported a 34% incidence of CMV infection, whereas our population exhibited a lower rate of 6.3%. ^16^ This may be due to our patients being on CMV prophylaxis for six months post-transplant.

In our study, the prevalence of post-transplant thrombocytopenia (PTT) is 31.2%, which is in line with Xie *et al*. ^28^ and Heaf *et al*. ^29^ where the prevalence was found to be 33.9% and 30%, respectively within the first year of transplantation. Previous studies have implicated immunosuppression regimens, including Grafalon and ATG, as causative agents. ^20^^,^^30^ In contrast, our observations indicated that infection was the primary trigger. ATG/Grafalon use was significantly lower in our patients. In addition, most of our patients with lower socioeconomic status were prone to infections.

We observed post-transplant pancytopenia (PTP) in 15.1% of the patients, and the major cause was drug-induced, which is consistent with previous studies. ^31^ Erythrocytosis was reported in 17.9% and it had male predominance, which was consistent with reports from Friman *et al*. and Kessler. ^32^^,^^33^

In the present study, 30 patients were identified with allograft dysfunction, including SGF/DGF and acute rejection. Among these, 19 graft rejections were observed, which aligns with the findings of previous studies. ^34^^,^^35^ Compared with our report, Talwar *et al*. reported a lower incidence of DGF (only 6%). Furthermore, in our cohort, graft dysfunction was higher in patients with severe anemia in the first week after transplantation. Consistent with this, Chhabra *et al*. reported a significant association between graft loss and PTA at three months. PTA-mediated allograft rejection/dysfunction is caused by limited oxygen delivery to the tubular interstitium. Hypoxia generates reactive oxygen species that cause oxidative insult to the renal tissues and mediate the release of pro-inflammatory cytokines, leading to the recruitment of inflammatory cells to the interstitium. ^36^

To assess graft function, we compared the early and late serum creatinine and eGFR levels in patients with post-transplant anemia at six and 12 months. Although patients with PTA had higher serum creatinine levels than the non-anemic group, the difference was not statistically significant and was similar in patients with and without PTA in our study. This was most likely due to the small sample size. In contrast, Molnar *et al*. studied 938 renal transplant recipients and showed a statistically significant reduction in renal function in patients with PTA. ^37^

### Limitations

The major limitation of our study was the small sample size, and the post-transplantation follow-up was limited to one year. This was a retrospective single-center study. Furthermore, we did not analyze the association between PTA and mortality or the severity of anemia and graft failure. Additionally, the young recipient age in our study may limit the genelizability to other populations. We did not perform iron studies or measure erythropoietin levels in our population.

## Conclusions

Haematological abnormalities are common in post-RT patients. PTA is the most frequent abnormality, and it improves over time. There was a significant improvement in hemoglobin levels at one, six, and 12 months post-transplant compared to pre-transplant **.** Leukopenia, thrombocytopenia, pancytopenia, and post-transplant erythrocytosis are other common haematological abnormalities. Leucopenia and pancytopenia was primarily drug-induced, and thrombocytopenia was frequently caused by infections in our cohort. Additionally, severe post-transplant anemia was significantly associated with graft dysfunction in the first week post-transplant. However, PTA did not show any association with graft function at six and twelve months post-transplantation.

## Data Availability

Figshare: This study contains the underlying data for ‘Post kidney transplant hematologic abnormalities and association of post-transplant anemia with graft function’ https://doi.org/10.6084/m9.figshare.25211441.v2. ^16^ Figshare: Extended data for ‘Post kidney transplant hematologic abnormalities and association of post-transplant anemia with graft function’, https://doi.org/10.6084/m9.figshare.24441268.v1. ^17^ This project contains the following extended data:
• Strobe checklist • Proforma • Protocol Data are available under the terms of the Creative Commons Attribution 4.0 International license (CC-BY 4.0)
