# Supplementary material for: Post kidney transplant hematologic abnormalities and association of post-transplant anemia with graft function
Source: F1000Res. 2024 Sep 26;13:241. Originally published 2024 Apr 2. [Version 2] doi: 10.12688/f1000research.144124.2 (PMC11503000; doi:10.12688/f1000research.144124.2)
Supplement: Peer review history

# Reviewer report: Reviewer response for version 2

**DOI:** 10.5256/f1000research.169665.r327104

**Published:** 2024-10-24

**Authors:** Dr Sonia Sharma

**Affiliations:** 1 Monash University, Clayton, Victoria, Australia

The authors have incorporated the suggested points in the revised manuscript. .All necessary changes have been made in accordance with the comments provided and there are no remaining concerns. Therefore, manuscript is ready to be indexed with no reservations.

Is the work clearly and accurately presented and does it cite the current literature?

Yes

If applicable, is the statistical analysis and its interpretation appropriate?

Yes

Are all the source data underlying the results available to ensure full reproducibility?

Yes

Is the study design appropriate and is the work technically sound?

Yes

Are the conclusions drawn adequately supported by the results?

Partly

Are sufficient details of methods and analysis provided to allow replication by others?

Yes

Reviewer Expertise:

Clinical Nephrology, Paediatric Nephrology, Kidney Transplantation,  MMF AUC work, Nephrotic syndrome, Tubular disorders

I confirm that I have read this submission and believe that I have an appropriate level of expertise to confirm that it is of an acceptable scientific standard.

# Reviewer report: Reviewer response for version 2

**DOI:** 10.5256/f1000research.169665.r327105

**Published:** 2024-10-16

**Authors:** Abraham Cohen-Bucay

**Affiliations:** 1 Massachusetts General Hospital Global Health, Boston, Massachusetts, USA

The authors successfully addressed all my concerns and recommendations. Therefore, I think the article is ready to be accepted without reservations from the scientific standpoint. However, there are some grammatical errors.

Is the work clearly and accurately presented and does it cite the current literature?

Yes

If applicable, is the statistical analysis and its interpretation appropriate?

Yes

Are all the source data underlying the results available to ensure full reproducibility?

Yes

Is the study design appropriate and is the work technically sound?

Yes

Are the conclusions drawn adequately supported by the results?

Yes

Are sufficient details of methods and analysis provided to allow replication by others?

Yes

Reviewer Expertise:

NA

I confirm that I have read this submission and believe that I have an appropriate level of expertise to confirm that it is of an acceptable scientific standard.

# Reviewer report: Reviewer response for version 1

**DOI:** 10.5256/f1000research.157873.r281382

**Published:** 2024-07-15

**Authors:** Dr Sonia Sharma

**Affiliations:** 1 Monash University, Clayton, Victoria, Australia

Good work done by the author. The research topic is important, and data from Asian countries are surely lacking.

 The study design is appropriate, and sufficient details are given in the method section. 

 However, in the result section, some details are required to make it more informative for indexing:

 1. High prevalence shown at one month and in the subsequent time period. It is required, therefore, if there were any associated ongoing etiologies. Related discussions and comments on previous data are also required later in the relevant section (discussion part). 

 2. What was the severity of anemia at three months, six months, and one year? 

 3. About Leukopenia and thrombocytopenia: At what stage of transplant were both noted?  For Leukopenia, 90% of study patients were shown to have mycophenolate mofetil association. What dose of mycophenolate was given if there were other side effects of the drug at the same time. 

 4. Were there any differences in parameters between cadaveric and live donors? 

 Minor: 

 1. The text and tables provide almost similar information. Either must be modified to avoid repetition, and better informative tables are required. 

 2. Figure 3. Clarity of message is required related to the proportion of patients. 

 3. Correction is required for the last line of the conclusion.

Is the work clearly and accurately presented and does it cite the current literature?

Yes

If applicable, is the statistical analysis and its interpretation appropriate?

Yes

Are all the source data underlying the results available to ensure full reproducibility?

Yes

Is the study design appropriate and is the work technically sound?

Yes

Are the conclusions drawn adequately supported by the results?

Partly

Are sufficient details of methods and analysis provided to allow replication by others?

Yes

Reviewer Expertise:

Clinical Nephrology, Paediatric Nephrology, Kidney Transplantation MMF AUC work, Nephrotic syndrome, Tubular disorders

I confirm that I have read this submission and believe that I have an appropriate level of expertise to confirm that it is of an acceptable scientific standard, however I have significant reservations, as outlined above.

# Response

**Published:** 2024-09-04

**Authors:** Shankar Prasad Nagaraju

**Affiliations:** Nephrology, Manipal Academy of Higher Education, Manipal, Karnataka, India

We would like to thank the editorial team of F1000 research for bringing in expert reviewers to provide valuable feedback. We are also extremely grateful to the reviewers for their valuable comments. We have revised our manuscript incorporating the changes suggested by reviewers

**Comment 1: High prevalence shown at one month and in the subsequent time period. It is required, therefore, if there were any associated ongoing etiologies. Related discussions and comments on previous data are also required later in the relevant section (discussion part)**

 Response: Dear Madam, Thank you for your valuable input on improving our manuscript.

 Since most PTA are due to surgical blood loss, we didn’t evaluate in detail for PTA in all patients. As suggested we have included and discussed the causes for post-transplant anaemia in discussion section.

**Comment 2: What was the severity of anemia at three months, six months, and one year?**

 Response: Thank you for the suggestion, which helped us refine our manuscript. We have stratified PTA severity at each time points into three categories (severe, mild, no anemia) in **Table 3 (Page No.10, Line No.127)**

**Comment 3: About Leukopenia and thrombocytopenia: At what stage of transplant were both noted?  For Leukopenia, 90% of study patients were shown to have mycophenolate mofetil association. What dose of mycophenolate was given if there were other side effects of the drug at the same time**

 Response: Sincere thanks for your suggestion. The presence or absence of leukopenia and thrombocytopenia were defined at post-transplant time points of <6 months and >6 months. The same has been defined in the methods section. **(Page No.8, Line No.105-109)**.

 During the pancytopenia episodes Valgancyclovir and/or trimethoprim/sulphamethoxozole were also stopped in addition to mycophenolate mofetil. ATG was given to 20.7% of patients as induction. Hence, as suggested by both the reviewers we have revised the manuscript. Instead of implying that the cytopenia was due to mycophenolate mofetil alone, we have mentioned that it is drug induced cytopenia. We sincerely thank both the reviewers for bringing this to our notice and helping us improving the manuscript.

**Comments 4: Were there any differences in parameters between cadaveric and live donors?**

 Response: Dear Madam, Thank you for this query. As 85.8% of the recipients received the kidney from live donors, the difference in the parameters between the cadaveric and live donors was not analysed.

**Minor**

**Comment 5: The text and tables provide almost similar information. Either must be modified to avoid repetition, and better informative tables are required**

 Response: Dear Madam, Thank you for the suggestion. The text has been suitably modified.

**Comment 6: Figure 3. Clarity of message is required related to the proportion of patients.**

 Response: Sincere thanks for the suggestion. We have revised the graph incorporating the proportion of patients **(Page No.11, Figure 3).**

**Comment 7: Correction is required for the last line of the conclusion**

 Response: Thank you for bringing this to our notice. This part has been modified **(Page No.14, Line No.285-286).**

# Reviewer report: Reviewer response for version 1

**DOI:** 10.5256/f1000research.157873.r281377

**Published:** 2024-06-11

**Authors:** Abraham Cohen-Bucay

**Affiliations:** 1 Massachusetts General Hospital Global Health, Boston, Massachusetts, USA

This single-center retrospective study examines the incidence of hematologic abnormalities in the first year post-transplant. Although not entirely novel, as some of the results are well-known (e.g., the high incidence of anemia, leukopenia, and thrombocytopenia), this report still contributes valuable information to the transplant community. However, several points should be addressed to enhance the manuscript:

  

1. Baseline Anemia Data: It would be beneficial to provide baseline anemia data pre-transplant. Although the mean hemoglobin level is provided, the percentage of patients with anemia is not. Given the post-transplant anemia incidence of 98% at one week, it is likely that the pre-transplant anemia incidence is similarly high
2. Anemia Definition and stratification: The study defines anemia as hemoglobin levels <12 g/dL in women and <13 g/dL in men. However, this definition is unrealistic for early post-transplant patients, given the target on dialysis is only >10 g/dL with erythropoietin treatment. Therefore, It would be informative to stratify the incidence of anemia severity: <10 g/dL, 10-12/13 g/dL, and no anemia (>12/13 g/dL)
3. Details on leukopenia: Further details regarding leukopenia would be informative. Specifically, distinguishing between lymphopenia and neutropenia, the number of patients with neutropenia, and whether any required G-CSF treatment for neutropenia
4. Timing of cytopenia definition: Please clarify if leukopenia and thrombocytopenia were defined at specific time points during follow-up or if any cytopenia (even transient) qualified a patient as having these conditions.
5. Valganciclovir and trimethoprim/sulfamethoxazole are known to cause leukopenia and bone marrow suppression. How many patients were on these medications, and were any of these medications needed to be discontinued due to leukopenia or other cytopenias?
6. Did any patients have thrombotic microangiopathy, which could explain the observed thrombocytopenia and anemia (hemolysis)?
7. Limitations: I concur with the limitations noted by the authors. Additionally, the young recipient age in your study may limit the generalizability to other populations.
8. Causes of cytopenias: The manuscript notes that most cytopenias were attributed to mycophenolate use rather than ATG, due to the low ATG dose. How did you reach the diagnosis of mycophenolate-induced cytopenia? How were other potential causes ruled out? Was the diagnosis of mycophenolate-induced cytopenia made by the treating clinician, or were hematology consultations sought?
9. Mycophenolate dosing: Please provide the initial dose of mycophenolate used at the study's outset, and detail any dose adjustments made due to cytopenias/anemia, including the extent of such reductions.

Is the work clearly and accurately presented and does it cite the current literature?

Yes

If applicable, is the statistical analysis and its interpretation appropriate?

Yes

Are all the source data underlying the results available to ensure full reproducibility?

Yes

Is the study design appropriate and is the work technically sound?

Yes

Are the conclusions drawn adequately supported by the results?

Yes

Are sufficient details of methods and analysis provided to allow replication by others?

Yes

Reviewer Expertise:

kidney transplantation

I confirm that I have read this submission and believe that I have an appropriate level of expertise to confirm that it is of an acceptable scientific standard, however I have significant reservations, as outlined above.

# Response

**Published:** 2024-09-04

**Authors:** Shankar Prasad Nagaraju

**Affiliations:** Nephrology, Manipal Academy of Higher Education, Manipal, Karnataka, India

We thank the editorial team of F1000 research for bringing in expert reviewers to provide valuable feedback. We are also extremely grateful to the reviewers for their valuable comments. We have revised our manuscript to incorporate the changes suggested by reviewers.

**Comment 1: Baseline Anemia Data: It would be beneficial to provide baseline anemia data pre-transplant. Although the mean hemoglobin level is provided, the percentage of patients with anemia is not. Given the post-transplant anemia incidence of 98% at one week, it is likely that the pre-transplant anemia incidence is similarly high**

 Response: Dear Sir, Many thanks for your suggestion to improve our manuscript. We have incorporated the percentage of pre-transplant anaemia as suggested in the

 result section. **(Page No.9, Line No.119)**

**Comment 2: Anemia Definition and stratification: The study defines anemia as hemoglobin levels <12 g/dL in women and <13 g/dL in men. However, this definition is unrealistic for early post-transplant patients, given the target on dialysis is only >10 g/dL with erythropoietin treatment. Therefore, It would be informative to stratify the incidence of anaemia severity: <10 g/dL, 10-12/13 g/dL, and no anaemia (>12/13 g/dL)**

 Response: Thank you for bringing this to our attention. Based on your feedback, we have revised manuscript to more accurately reflect the clinical context of PTA. Specifically, we have categorized the severity of PTA in **Table 3** as suggested into three distinct groups as no anaemia (>12/13 g/dL)

 <10 g/dL, mild anaemia   10-12/13 g/dL, and severe (<10 g/dL). **(Page No.10, Line No.127)**

**Comment 3: Details on leukopenia: Further details regarding leukopenia would be informative. Specifically, distinguishing between lymphopenia and neutropenia, the number of patients with neutropenia, and whether any required G-CSF treatment for neutropenia**

 Response: Many thanks for the suggestion. We have added information regarding neutropenia and number of patients who required G-CSF treatment for neutropenia in the result section **(Page No.11, Line No.164-168)**

**Comment 4: Timing of cytopenia definition: Please clarify if leukopenia and thrombocytopenia were defined at specific time points during follow-up or if any cytopenia (even transient) qualified a patient as having these conditions**

 Response: Sincere thanks for your suggestion. The presence or absence of leukopenia and thrombocytopenia were defined at post-transplant time points of <6 months and >6 months. The same has been defined in the methods section **(Page No.8, Line No.79-80)**. The same has been added in the results section too **(Page No.11, Line No.164).**

**Comment 5: Valgancyclovir and trimethoprim/sulfamethoxazole are known to cause leukopenia and bone marrow suppression. How many patients were on these medications, and were any of these medications needed to be discontinued due to leukopenia or other cytopenia?**

 Response: Dear Sir, Thank you for the valuable input. Our patients were on Valgancyclovir prophylaxis until first 6 months and the trimethoprim sulfamethoxazole up to 12 months after kidney transplantation. They were also stopped during leukopenia. So we have revised the section on leukopenia in the manuscript with the details and as suggested instead of implying that mycophenolate mofetil as cause of leukopenia we have mentioned it as drug induced leukopenia **(Page No.12, Line No.180-186).**

**Comment 6: Did any patients have thrombotic microangiopathy, which could explain the observed thrombocytopenia and anemia (hemolysis)?**

 Response: Dear Sir, Many thanks for the suggestion. None of our patients had TMA As indicated, we have incorporated the same in the result section in the manuscript **(Page No.9, Line No.124-125).**

**Comment 7: I concur with the limitations noted by the authors. Additionally, the young recipient age in your study may limit the generalizability to other populations.**

 Response: Dear Sir, Thank you for the input. The same has been added in the limitation section as suggested **(Page No.14, Line No.274-275)**

**Comment 8: Causes of cytopenia: The manuscript notes that most cytopenias were attributed to mycophenolate use rather than ATG, due to the low ATG dose. How did you reach the diagnosis of mycophenolate-induced cytopenia? How were other potential causes ruled out? Was the diagnosis of mycophenolate-induced cytopenia made by the treating clinician, or were hematology consultations sought?**

 Response: Dear Sir, Thank you for the valuable suggestions and insights to improve the manuscript. ATG may have a superimposed impact in patients where it has been administered. Additionally, when pancytopenia was present, other medications like Valgancyclovir and trimethoprim sulfamethoxazole were also stopped. All patients were tested for CMV RNA PCR during the pancytopenia episodes. The manuscript has been revised and mentioned as drug induced cytopenia as suggested instead of mycophenolate induced cytopenia. We thank the reviewer for bringing this into our notice and guiding us in revising the manuscript. The decision was made by Nephrologist and clinical hematology consultation was not sought due to non-availability of haematologist at our institute during the study period. **(Page No.12, Line No.180 and 187).**

**Comment 9: Mycophenolate dosing: Please provide the initial dose of mycophenolate used at the study's outset, and detail any dose adjustments made due to cytopenias/anemia, including the extent of such reductions**

 Response: The mycophenolate dose used varied from 1.5-2.00 gm as induction based on the body weight and nephrologist clinical decision. The dose was reduced to 0.5-1.0 g per day after the cytopenia episodes and was adjusted further on follow-up on case to case basis as per etiology. The same has been incorporated in the manuscript **(Page No.8, Line No.105-109)**
